# Serum Zinc Status and Its Association with Allergic Sensitization: The Fifth Korea National Health and Nutrition Examination Survey

**DOI:** 10.1038/s41598-017-13068-x

**Published:** 2017-10-03

**Authors:** Hyun-Min Seo, Yeong Ho Kim, Ji Hyun Lee, Joung Soo Kim, Young Min Park, Jun Young Lee

**Affiliations:** 1 Department of Dermatology, Hanyang University Guri Hospital, Guri, Korea; 2 Department of Dermatology, Seoul St. Mary’s Hospital, College of Medicine, The Catholic University of Korea, Seoul, Korea

## Abstract

Zinc (Zn) is an essential trace element that plays important roles in the immune system. There is little known about the role of trace elements in allergic diseases, and previous reports have shown conflicting results. The aim of this study was to investigate the relationship between serum Zn levels and total or allergen-specific immunoglobulin E (IgE) levels. The initial candidates for this study were those who participated in the 5^th^ Korean National Health and Nutrition Examination Survey 2010 (n = 8,958), and 1,867 adults who had serum total and allergen specific-IgE levels measured were included. Upon adjusting for covariates, mean total IgE, *Dermatophagoides farinae* and dog-specific IgE levels increased significantly as the Zn levels decrease from the highest to the lowest quartile (*p* = 0.009, 0.004, and < 0.001, respectively). The multiple logistic regression analyses showed significant negative linear correlations between serum Zn levels and total, *D. farinae-*, cockroach-, and dog-specific IgE levels (p-value for linear trend = 0.004, 0.006, 0.027, and < 0.001, respectively). This study demonstrated that total/allergen specific IgE and Zn levels are significantly inversely related.

## Introduction

Type I hypersensitivity clinically manifests in a variety of allergic diseases such as atopic dermatitis, asthma, allergic rhinitis, allergic conjunctivitis, food allergies and specific types of urticaria^1^. Immunoglobulin E (IgE) has a central role in type I hypersensitivity, which reflects the sensitization of mast cells by allergen-specific IgE antibodies bound to their high-affinity receptors (FcεRI)^2^.

Zinc (Zn) is an essential trace element that plays important roles in the immune system, from contributing to the skin barrier to gene regulation in lymphocytes^3^. Zn is essential for FcεRI-mediated cytokine production in mast cell and transcription of IL-6 and TNF-α mRNA^4^. Zn is also known to function as an antioxidant and stabilize cell membranes^3^. Zn deficiency affects about 2.2 billion people around the world and as much as 25% of the world’s population is at risk^5^. A Korean study of 245 pregnant women showed that low Zn status is highly prevalent (76.3%)^6^. The clinical manifestations of Zn deficiency include alopecia, acrodermatitis enteropathica, diarrhea, emotional disorders, weight loss, dysfunction of cell-mediated immunity and neurological disorders^5,7^.

There is little known about the role of trace elements in allergic diseases and previous reports have shown conflicting results. There have been some reports of association between decreased Zn levels and asthma and atopic dermatitis^8–10^, whereas other studies have not shown a significant association^11,12^. There have also been a few reports on the effects of Zn supplementation in asthma and atopic dermatitis^7,13,14^. To date, there has been no study of the association of IgE levels with Zn levels. The aim of this study was to investigate the relationship between serum Zn levels and total or allergen-specific IgE levels using the Korea National Health and Nutrition Examination Survey (KNHANES) databases.

## Methods

### Study design and database

**Figure 1:**
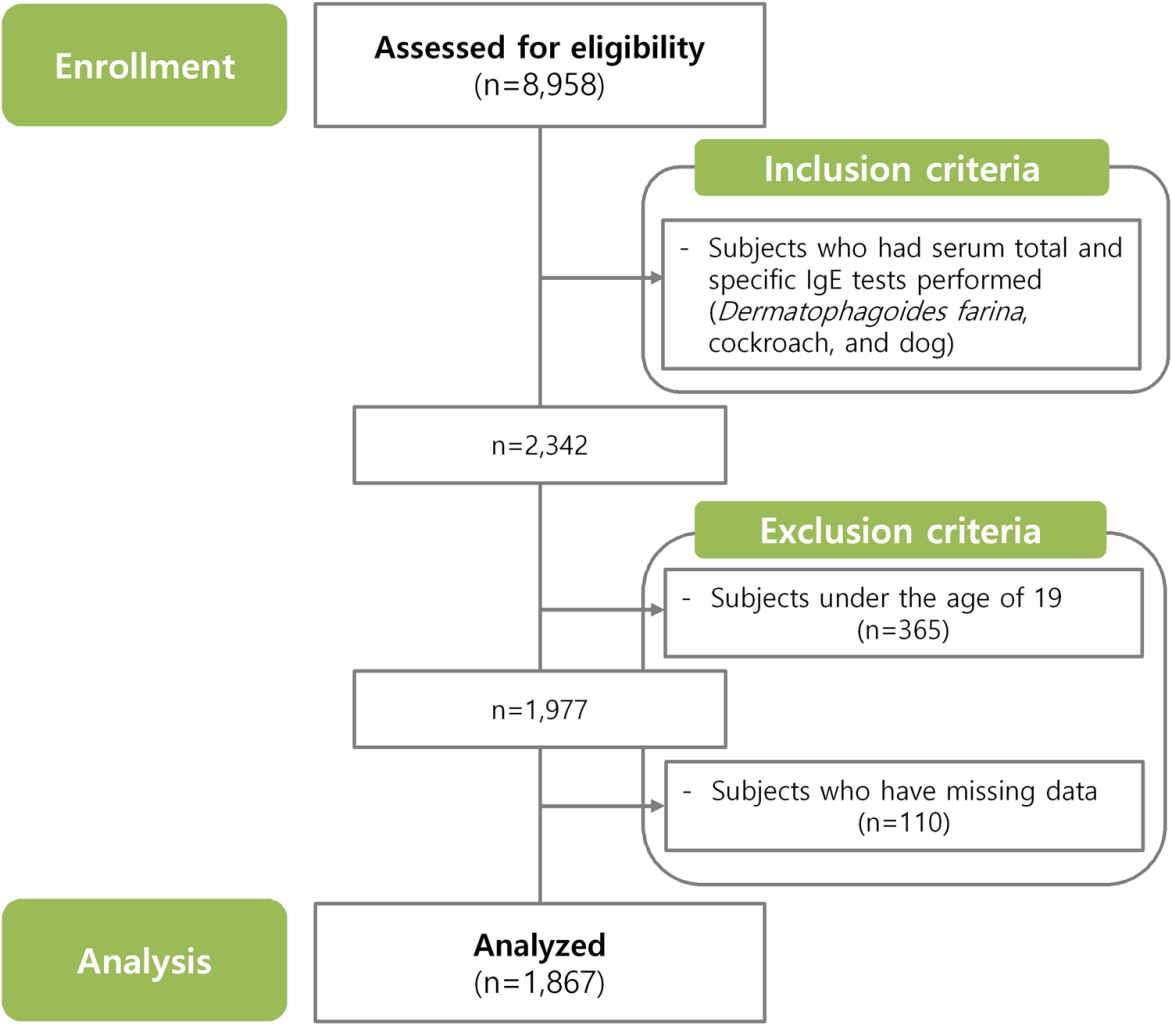
Flowchart of the study.

This study was a retrospective cross-sectional study using the 5^th^ KNHANES conducted by the Korea Centers for Disease Control and Prevention from 2010 January to 2012 December. However, since serum Zn and IgE tests were conducted only in 2010, the database from 2010 January to 2010 December was utilized in this study. Selection of the household units and participants for the 5^th^ KNHAES was made based on a complex, multi-stage, probability sampling design. KNHANES collects a number of variables regarding the participants’ demographic, social, health and nutritional statuses using three component surveys: the health interview, the health examination and the nutrition survey. The surveys collect detailed information on anthropometric measures, health behaviors, socioeconomic status, healthcare utilization, quality of life, biochemical profiles using fasting blood serum and urine, measures for dental health, vision, hearing and bone density, X-ray test results, food intake and dietary behavior. Blood and urine tests are performed in participants 10 years of age or older. Other details about the 5^th^ KNHANES have been described previously^15^. The initial candidates for this study were those who participated in the 5^th^ KNHANES 2010 (n = 8,958). We selected subjects who had serum total and allergen-specific IgE (*Dermatophagoides farinae*, cockroach and dog) tests performed (n = 2,342). Then, we excluded the subjects under the age of 19 (n = 365) and those who had missing data (n = 110). Finally, a total of 1,867 subjects were included in this study (Fig. 1). This study was approved by the Korean Ministry of Health and Welfare (2010 – 02CON-21-C) and was conducted per the principles of the Declaration of Helsinki.

### Anthropometric measurements

Height (cm) was measured by medical stadiometer (Seca 225, Seca, German) with the subject facing directly forward with the shoes off, feet together, arms by the sides, and the heels, buttocks, and upper back in contact with the wall. Weight (kg) were measured by digital medical weight scale (GL-6000–20, G-Tech International co., South Korea) while they were wearing light clothes. Results were expressed by rounded to the second decimal place and recorded by a trained examiner. To maintain the accuracy of the measurements, the instruments have been replaced with newly calibrated devices every year. Body mass index (BMI) was calculated by dividing weight (kg) by height squared (m^2^). Metabolic syndrome was defined as having at least three of the following; (1) central obesity: waist circumference ≥ 90 cm (Asian male), ≥ 85 cm (Asian female), (2) dyslipidemia: triglyceride ≥ 150 mg/dl, (3) dyslipidemia: high-density lipoprotein (HDL) < 40 mg/dL (male), < 50 mg/dL (female), (4) blood pressure ≥ 130/85 mmHg (or treated for hypertension), (5) fasting plasma glucose ≥ 100 mg/dl^16,17^.

### Variables

The population was divided according to residential area: rural or urban. The study subjects’ socioeconomic factors were surveyed by a self-administered questionnaire. The household income was estimated by standardization methods for classifications based on the national standard income level. The household income was categorized into four quartiles with very low (0 to 25^th^ percentile), low (25^th^ to 50^th^ percentile), medium (50^th^ to 75^th^ percentile) and high (75^th^ to 100^th^ percentile). All subjects completed a survey to determine their smoking history (never, former or current) and alcohol consumption habits (glasses per day in a recent month). Comorbidities, including atopic dermatitis, asthma and allergic rhinitis, were identified with a self-reported questionnaire about past diagnoses. Other details have been described elsewhere^15^.

### Biochemical analysis

Peripheral blood samples were obtained after an 8-h fast, and then were immediately processed and transported to a central laboratory (Neodin Medical Institute, Seoul, South Korea). All blood samples were analyzed within 24 h of collection. Serum Zn levels were determined by inductively coupled plasma mass spectrometry (ICP-MS, PerkinElmer Inc., Waltham, MA, USA). In previous studies, low serum zinc levels were analyzed using the 1^st^ tertile or quartile^18,19^. In our subjects, the median and 25^th^ quartile serum Zn levels were 134.0 µg/dL and 117.0 µg/dL, respectively, so the authors defined hypozincemia as a plasma Zn level below 120 µg/dL for comparison of baseline and clinical characteristics. Total serum IgE and allergen-specific IgE (*D. farina*, cockroach and dog) levels were examined by immunoradiometric assay (1470 WIZARD gamma-Counter, PerkinElmer Inc.). An increase in serum total IgE levels was defined as levels greater than 100 kU/L, and subjects with allergen-specific IgE levels of 0.35 kU/L or more were defined as being sensitized to that specific allergen^20^.

### Statistical analyses

Continuous variables were presented as means with their respective standard deviation. To analyze the baseline characteristics of subjects, t-tests and chi-square tests and were used for continuous and categorical variables, respectively. Comparisons of mean total and allergen-specific IgE levels according to the quartile of the serum Zn levels were analyzed via analysis of covariates (ANCOVA). Model 1 was non-adjusted; model 2 was adjusted for age and sex; model 3 was adjusted for age, sex, BMI, smoking, drinking, exercise, education, income, metabolic syndrome and vitamin D level. We divided serum Zn levels into 4 groups according to quartile ranges.

We also used multivariable logistic regression analyses to examine the associations between serum Zn levels and total and allergen-specific IgE levels, adjusting for age, sex, BMI, smoking, drinking, exercise, education, income, metabolic syndrome and vitamin D level. Statistical significance was considered present when the *P* value was less than 0.05. Data were analyzed using SAS version 9.4 (SAS Institute, Cary, NC, USA).

## Results

### Demographic and clinical characteristics

This study included a total of 1,867 subjects, and baseline demographic and clinical characteristics are summarized in Table 1. Among the 1,867 subjects, 554 subjects were classified as hypozincemia. The hypozincemia group had a lower percentage of males (35.6%, *p* < 0.001), current smokers (19.1%, *p* = 0.002), and lower overall alcohol consumption (53.8%, *p* = 0.002) compared to subjects without hypozincemia. There were significant differences in mean BMI (23.4 ± 3.3 kg/m^2^, *p* = 0.025), total cholesterol (184.4 ± 37.8 mg/dL, *p* = 0.023), triglyceride (123.4 ± 105.6 mg/dL, *p* = 0.016), HDL (50.2 ± 11.5 mg/dL, *p* < 0.001), low-density lipoprotein (LDL, 109.1 ± 32.3 mg/dL, *p* = 0.004) and vitamin D (16.7 ± 5.9 ng/mL, *p* < 0.001) levels between subjects with hypozincemia and those without hypozincemia. Regarding allergic sensitization, subjects with hypozincemia were most sensitized to *D. farina* (37.9%), followed by cockroaches (19.0%) and dogs (6.7%). The subjects without hypozincemia showed comparable results (38.4, 20.6 and 4.7%, respectively), and in both groups 44.6% of patients showed increased serum total IgE levels.

**Table 1:** Baseline and clinical characteristics of the study population.

Characteristics		Subjects without hypozincemia (≥120 µg/dL) n = 1,313	Subjects with hypozincemia (<120 µg/dL) n = 554	p value
Age, years (SD)		44.9 (14.5)	44.8 (15.3)	0.870
Male, n (%)		721 (54.9)	197 (35.6)	<0.001
BMI, kg/m^2^ (SD)		23.7 (3.4)	23.4 (3.3)	0.025
House income				0.942
	Very low (0~25%), n (%)	205 (15.6)	88 (15.9)	
	Low (25~50%), n (%)	357 (27.2)	151 (27.3)	
	Moderate (50~75%), n (%)	373 (28.4)	150 (27.1)	
	High (75~100%), n (%)	378 (28.8)	165 (29.8)	
Region				0.996
	Urban, n (%)	614 (46.8)	259 (46.8)	
	Rural, n (%)	699 (53.2)	295 (53.2)	
Smoking				0.002
	Non-smoker, n (%)	661 (50.3)	340 (61.4)	
	Ex-smoker, n (%)	276 (21.0)	108 (19.5)	
	Current smoker, n (%)	376 (28.6)	106 (19.1)	
Alcohol consumption (at least once a month), n (%)		807 (61.5)	298 (53.8)	0.002
Asthma, n (%)		36 (2.7)	15 (2.7)	0.967
Atopic dermatitis, n (%)		40 (3.0)	15 (2.7)	0.692
Allergic rhinitis, n (%)		201 (15.3)	80 (14.4)	0.632
Metabolic syndrome, n (%)		306 (23.3)	112 (20.2)	0.145
Systolic blood pressure, mmHg (SD)		117.4 (16.7)	116.4 (16.7)	0.210
Diastolic blood pressure, mmHg (SD)		75.2 (10.5)	74.6 (10.2)	0.283
Fasting glucose				0.249
Within normal range (<100 mg/dL), n (%)		1003 (76.4)	404 (72.9)	
Impaired fasting glucose (100~125 mg/dL), n (%)		238 (18.1)	112 (20.2)	
Elevated fasting glucose(≥126 mg/dL), n (%)		72 (5.5)	38 (6.9)	
Total cholesterol, mg/dL (SD)		188.6 (36.9)	184.4 (37.8)	0.023
Triglyceride, mg/dL (SD)		138.3 (127.7)	123.4 (105.6)	0.016
HDL, mg/dL (SD)		48.0 (11.1)	50.2 (11.5)	<0.001
LDL, mg/dL (SD)		113.8 (31.6)	109.1 (32.3)	0.004
Increased serum total IgE, n (%)		585 (44.6)	247 (44.6)	0.990
Sensitization to specific allergen				
	*Dermatophagoides farinae*, n (%)	504 (38.4)	210 (37.9)	0.846
	Cockroaches, n (%)	271 (20.6)	105 (19.0)	0.406
	Dogs, n (%)	62 (4.7)	37 (6.7)	0.085
Serum Zn, µg/dL (SD)		149.6 (24.7)	105.4 (10.9)	<0.001
Vitamin D, ng/mL (SD)		18.6 (6.6)	16.7 (5.9)	<0.001

BMI, body mass index; HDL, high-density lipoprotein; IgE, immunoglobulin E; LDL, low-density lipoprotein; SD, standard deviation; Zn, zinc.

### ***A***ssociation between serum Zn levels and allergic sensitization

The 25^th^ percentile, median, and 75^th^ percentile value of serum Zn levels were 31.0, 82.0, and 250.0 kU/L, respectively. Estimated mean serum total and allergen-specific IgE levels between Q1 and Q4 both before and after adjusting for covariates are shown in Table 2. Before adjusting for covariates, total and dog-specific IgE levels tended to increase as Zn levels decreased from Q4 to Q1, and these associations were statistically significant (*p* = 0.039 and < 0.001, respectively). Upon adjusting for covariates (models 2 and 3), the negative correlation between serum Zn levels and total and allergen-specific IgE levels was more pronounced. As Zn levels decreased from Q4 to Q1, mean total IgE, *D. farinae* and dog-specific IgE levels increased significantly (model 2: *p* = 0.035, 0.010, and < 0.001, respectively; model 3: *p* = 0.009, 0.004, and < 0.001, respectively). However, the relationship between cockroach-specific IgE levels and serum Zn levels was not statistically significant, either before or after adjusting for covariates.

**Table 2:** Association between serum zinc levels and allergic sensitization.

		Serum Zn level				*p* value
		Q1	Q2	Q3	Q4	
Total IgE (U/mL)	Model 1	111.6 (93.8–132.7)	93.8 (78.9–111.6)	80.3 (66.0–97.6)	83.9 (71.4–98.5)	0.039
	Model 2	110.8 (93.1–131.9)	94.3 (79.7–111.5)	78.9 (65.4–95.3)	84.5 (72.5–98.5)	0.035
	Model 3	111.4 (93.9–132.1)	92.0 (77.6–109.0)	76.7 (63.7–92.3)	79.1 (68.4–91.6)	0.009
*Dermatophagoides farinae*	Model 1	0.30 (0.23–0.40)	0.23 (0.17–0.31)	0.17 (0.13–0.23)	0.23 (0.18–0.30)	0.052
	Model 2	0.32 (0.24–0.42)	0.23 (0.17–0.31)	0.17 (0.13–0.22)	0.21 (0.16–0.28)	0.010
	Model 3	0.33 (0.25–0.45)	0.23 (0.17–0.31)	0.17 (0.13–0.22)	0.19 (0.15–0.25)	0.004
Cockroach	Model 1	0.11 (0.088–0.137)	0.10 (0.09–0.12)	0.09 (0.07–0.12)	0.09 (0.08–0.12)	0.721
	Model 2	0.11 (0.089–0.138)	0.10 (0.09–0.12)	0.09 (0.07–0.11)	0.09 (0.08–0.12)	0.633
	Model 3	0.11 (0.092–0.136)	0.10 (0.08–0.11)	0.09 (0.07–0.11)	0.09 (0.08–0.11)	0.429
Dog	Model 1	0.04 (0.035–0.051)	0.03 (0.03–0.04)	0.03 (0.02–0.03)	0.03 (0.02–0.03)	<0.001
	Model 2	0.04 (0.036–0.052)	0.03 (0.03–0.04)	0.03 (0.02–0.03)	0.03 (0.02–0.03)	<0.001
	Model 3	0.04 (0.036–0.053)	0.03 (0.03–0.04)	0.03 (0.02–0.03)	0.02 (0.02–0.03)	<0.001

IgE, immunoglobulin E; Q1, 1^st^ quartile; Q2, 2^nd^ quartile; Q3, 3^rd^ quartile; Q4, 4^th^ quartile. ^*^Model 1: not adjusted; Model 2: adjusted for age and sex; Model 3: adjusted for age, sex, body mass index, smoking, alcohol consumption, income, metabolic syndrome and serum vitamin D level.

### Effect of serum Zn levels on the odds ratios for allergic sensitization

**Figure 2:**
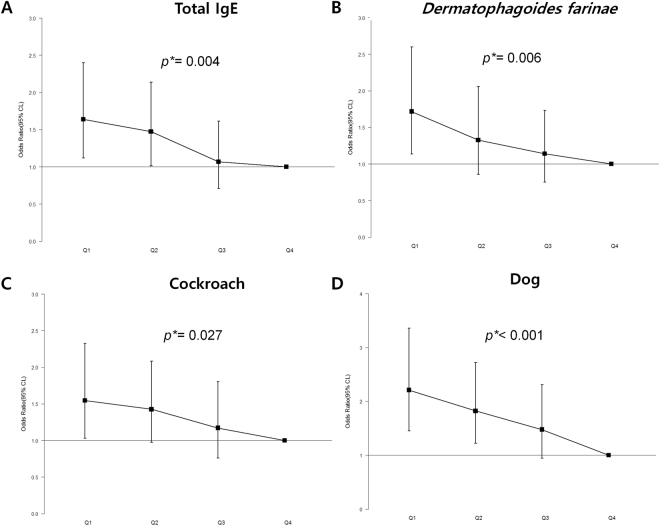
Effects of serum Zn levels on the odds ratios for allergic sensitization. CI, confidence interval; IgE, immunoglobulin E; Q1, 1^st^ quartile; Q2, 2^nd^ quartile; Q3, 3^rd^ quartile; Q4, 4^th^; Q, quartile. ^*^p-value for linear trend.

Multivariable logistic regression analyses were performed for quartiles based on serum Zn levels to examine the association of serum Zn level with both total and allergen-specific IgE levels. Upon adjustment for age, sex, BMI, smoking, drinking, exercise, education, income, metabolic syndrome and vitamin D level, the trend analyses showed significant negative linear correlations between serum Zn levels and total, *D. farina-*, cockroach-, and dog-specific IgE levels (p-value for linear trend = 0.004, 0.006, 0.027, and < 0.001, respectively) (Fig. 2).

## Discussion

This is the first study to evaluate the association between serum IgE levels and Zn levels in a general adult population of 1,867. Our study demonstrates that study subjects with increased total and allergen-specific IgE levels had significantly lower Zn levels. The trend analysis also showed significant negative linear correlations between total, *D. farinae-*, cockroach-, and dog-specific IgE levels and serum Zn levels.

Several conditions have been reported to be associated with Zn levels. Zn deficiency is clinically manifested in alopecia, acrodermatitis enteropathica, diarrhea, emotional disorders, weight loss, dysfunction of cell-mediated immunity and neurological disorders^5,7^. Recently, a study reported the associations between serum Zn levels and metabolic syndrome. They showed Serum Zn levels were negatively associated with elevated fasting glucose and positively associated with elevated triglycerides in men. They also showed that Zinc levels have a decreasing trend with increasing numbers of metabolic syndrome components in women with metabolic syndrome^19^. Therefore, we have included previously known factors in our analyses which may be associated with serum Zn levels. After adjusting the covariates, our results demonstrated that decreased serum Zn levels are associated with increased total IgE levels and allergic sensitization, including sensitization to *D. farinae*, cockroach and dog. There have been several reports investigating the relationship between Zn levels and allergic diseases, including allergic asthma and atopic dermatitis. Most recently, Nazila *et al*. reported that mean serum Zn levels in patients with allergic asthma were significantly lower than in healthy control subjects, although no correlation was found between Zn levels and disease severity^10^. In a different study on atopic dermatitis, it was reported that erythrocyte Zn levels, which have been reported to be a better measure of mild Zn deficiency, were significantly lower in patients with atopic dermatitis when compared to in healthy control subjects^9^. On the other hand, David *et al*., who first suggested the association between serum zinc levels and atopic dermatitis in 1984, found no significant difference in a later article^11^. Although there have been conflicting results, there might be a link between serum Zn levels and allergic sensitization that is characterized by total and specific IgE.

There have been a few reports about the benefits of Zn supplements in managing allergic diseases. Two animal models show that mice fed with a zinc-deficient diet developed AD-like skin lesions^21,22^. These reports showed that serum IgE levels and the number of *S. aureus* on the skin surface were both increased in DS-*Nh* mice. In a study of children with atopic dermatitis, Zn supplementation for 8 weeks resulted in a significant increase in Zn levels in hair, and significant reductions in eczema area severity index, transepidermal water loss and visual analogue scales for pruritus when compared to healthy control subjects^7^. In a randomized, placebo-controlled trial in children with asthma, significant improvement in clinical symptoms and spirometry parameters was also observed in Zn supplementation groups^14^.

The exact role of Zn in allergic diseases remains unclear. Zn affects many aspects of immune system function, and is involved in FcεRI-induced mast cell activation^4^. FcεRI stimulation has been reported to trigger microtubular formation in a calcium-independent manner^23^. Zn has been shown to be involved in multiple steps of FcεRI-induced mast cell activation, and is required for degranulation and cytokine production^4^. Additionally, a complex interaction between Zn, cyclic nucleotide and nitric oxide signaling, inhibitors of κB kinase and inhibition of IL-1 receptor-associated kinase-1 counteracts the production of proinflammatory cytokines^24^. In light of these findings, Zn released from human mast cells may play a role in the regulation of the inflammation in allergic diseases.

Our study has several limitations. First, we did not analyze the relationship between serum Zn levels and disease, nor between total IgE/specific IgE levels and disease. Second, it remains unclear whether a single measurement of serum Zn levels accurately represents actual Zn status in the general population, and our analyses were not able to analyze causal relationships because of their cross-sectional nature. Prospectively designed studies are necessary to clarify the relationship between serum Zn levels and subsequent allergic sensitization. In spite of these limitations, this study has strength in its use of a comprehensive national database to represent the total Korean population, and in being the first to assess the association between serum Zn levels and allergic sensitization.

In conclusion, our findings demonstrated that increased total and allergen-specific IgE levels are significantly associated with decreased Zn levels. Future studies should clarify the role of Zn in the development of allergic sensitization.
